# A Novel QR Code–Based Solution for Secure Electronic Health Record Transfer in Venous Thromboembolism Home Rehabilitation Management: Algorithm Development and Validation

**DOI:** 10.2196/69230

**Published:** 2025-08-11

**Authors:** Changzhen Li, Zhigeng Jin, Fei Wang, Zheqi Zhang, Binbin Liu, Yutao Guo

**Affiliations:** 1 Technical Department, DrBreath Medical Technology Co, Ltd, Shanghai, China; 2 School of Information, Renmin University of China, Beijing, China; 3 Department of Pulmonary Vascular and Thrombotic Disease, Sixth Medical Center of Chinese PLA General Hospital, No.6 Fucheng Road, Haidian District, Beijing, 100048, China, 86 13810021492; 4 Chinese PLA Medical School, Beijing, China

**Keywords:** venous thromboembolism, home rehabilitation, electronic health record, QR code, secure data transmission, data compression, authenticated encryption, tokenization algorithm, data serialization, health information exchange, Avro

## Abstract

**Background:**

Venous thromboembolism (VTE) is a common vascular disorder requiring extended anticoagulation therapy postdischarge to reduce recurrence risk. Home rehabilitation management systems that use electronic health records from hospital care provide opportunities for continuous patient monitoring. However, transferring medical data from clinical to home settings raises significant concerns about privacy and security. Conventional methods such as manual data entry, optical character recognition, and dedicated data transmission lines face notable technical and operational challenges.

**Objective:**

This study aims to develop a QR code–based security transmission algorithm using Avro and byte pair encoding (BPE). The algorithm supports the secure creation and transfer of out-of-hospital health records by enabling patients to scan QR codes via a dedicated mobile app, ensuring data security and user privacy.

**Methods:**

Between January and October 2024, 300 hospitalized patients with VTE were recruited at the Sixth Medical Center of the Chinese PLA General Hospital. Post discharge, participants used a home rehabilitation app tailored for VTE management. The QR code–based security transmission algorithm was developed to securely transfer in-hospital electronic health records to the out-of-hospital app. It uses BPE, Avro, and Gzip for optimized data compression and uses ChaCha20 and BLAKE3 for encryption and authentication. Specifically, BPE tokenizes medical text, while Avro serializes JSON (JavaScript Object Notation) objects, contributing to data encryption. A proprietary tokenizer was trained, and compression efficiency was evaluated using a “Performance Benchmark Dataset.” Comparative analyses were conducted to assess the compression efficiency of JSON serialization methods (Avro and ASN.1 [Abstract Syntax Notation One]), and tokenization algorithms (BPE and unigram).

**Results:**

The dataset consisted of JSON files from 300 patients, averaging 240.1 fields per file (range 89‐623) and 7095 bytes in size (range 2748‐17,425 bytes). Using the BPE + Avro + Gzip algorithm, the average file size was reduced to 1048 bytes, achieving a compression ratio of 6.67. This was 1.82 times more efficient than traditional Gzip compression (average file size: 1907 bytes; compression ratio: 3.66; *P*<.001). For Chinese medical text tokenization, BPE outperformed unigram with a compression ratio of 4.68 versus 4.55 (*P*<.001). Avro and ASN.1 demonstrated comparable compression ratios of 2.57 and 2.59, respectively, when used alone (*P*=.30). However, Avro combined with BPE and Gzip significantly outperformed ASN.1, achieving compression ratios of 6.67 versus 5.21 (*P*<.001). Additionally, 84.7% (254/300) of patients needed to scan only 1 QR code, requiring an average of 3.1 seconds.

**Conclusions:**

The QR code–based security transmission algorithm using Avro and BPE efficiently compresses and transmits data in an encrypted manner and authenticates the identity of the scanning users, ensuring the privacy and security of medical data. Delivered as a software development kit, the algorithm offers straightforward implementation and usability, supporting its broad adoption across various applications.

## Introduction

Venous thromboembolism (VTE) is a leading cause of death and disability worldwide [1,2]. Patients with VTE require prolonged anticoagulant therapy after discharge to prevent the recurrence of thrombosis, and extended home rehabilitation management effectively reduces the incidence of VTE events [3]. To enhance the efficiency of home rehabilitation management for patients with VTE, we developed a home rehabilitation mobile health app to assist doctors in managing patients’ recovery outside the hospital. According to guidelines and consensus, home anticoagulation management for VTE is complex, requiring not only professional medical knowledge but also accurate patient information, such as coagulation indicators, biochemical markers (eg, liver, kidney, and heart function), underlying diseases, and bleeding risks [4]. Therefore, establishing comprehensive health records is the first step in effective home rehabilitation management. However, due to the multitude of information fields, manual entry by patients is difficult and leads to low user engagement.

Using patients’ electronic health records (EHRs) during hospitalization can improve the success rate of out-of-hospital health record creation [5]. Hospital Information Systems are typically deployed within internal (intranet) environments, while out-of-hospital services operate on public internet infrastructure. Industry standards such as HL7 Fast Healthcare Interoperability Resources have been proposed to enable cross-institutional data exchange. However, in practice, the deployment of Fast Healthcare Interoperability Resources or other application programming interface–based approaches faces multiple constraints. Their implementation depends on dedicated network connections via front-end machines or network gateways. This implementation approach introduces the following significant issues: (1) high hardware deployment and maintenance costs; (2) increased exposure of hospital intranets, elevating security risks; (3) low efficiency of cross-departmental collaboration, leading to slow service response; and (4) complete incompatibility with closed-network environments. Consequently, such protocols have not been widely adopted in domestic settings.

Some alternative approaches attempt to use optical character recognition on printed reports. However, the recognition accuracy (75.86%‐92.46%) is highly affected by scan quality and layout [6], generally requiring manual validation [7], making it difficult to scale for widespread use. Other solutions use QR code–based transmission [8-14], either transmitting in plaintext—posing leakage risks—or relying solely on traditional compression algorithms such as Gzip, which offer limited compression efficiency and fail to make full use of QR code capacity. In real-world scenarios, a single QR code typically holds only 500‐1500 bytes, further highlighting the importance of efficient encoding. Additionally, certain implementations require specialized screens and camera equipment, failing to balance medical privacy protection with end user convenience.

To address these challenges in cross-network medical information exchange, this study proposed a secure QR code–based transmission algorithm. The algorithm encrypts, compresses, and partitions JSON-formatted (JavaScript Object Notation) health records into multiple QR codes. Patients can safely, easily, and accurately complete the creation and transfer of out-of-hospital records simply by scanning the QR codes using a dedicated mobile health app.

To comprehensively demonstrate the advantages of the proposed solution in terms of recognition accuracy, data privacy, network exposure risk, and deployment cost, Table 1 presents a comparative analysis between this method and existing typical solutions (including optical character recognition–based solutions and dedicated channel solutions) in cross-network scenarios.

In this study, we have designed the algorithm framework in detail, described the compression and encryption processes, and constructed a training dataset. Finally, we validated the algorithm’s performance using real-world clinical data.

**Table 1.** Comparison of this proposal and existing medical information exchange solutions in cross-network scenarios.

Dimension	OCR-based^a^ solution	Dedicated channel (FHIR^b^ or other API^c^)	Proposed scheme
Accuracy	Low. Affected by image quality and layout; accuracy ranges from 75.86% to 92.46%, manual verification required	High. System-level integration with clear structure and minimal errors	High. Structured data embedded in QR code; stable decoding, unaffected by image quality
Data privacy	Low. Plaintext image transmission with high leakage risk	Medium-high. Relies on physical isolation and encryption; security depends on implementation	High. End-to-end encryption and authentication; supports zero-trust access
Network exposure risk	None. Typically offline image transmission, no network exposure	High. Requires open interfaces; increases attack surface and risk of intrusion	None. Offline QR transmission; no exposed network entry
Deployment cost	Low. No system changes needed, but high manual verification overhead	High. Requires gateway systems and dedicated channels; complex implementation and maintenance	Low. Lightweight SDK integration; high compatibility and easy deployment

a OCR: optical character recognition.

b FHIR: Fast Healthcare Interoperability Resources.

c API: application programming interface.

## Methods

### Algorithm Design

#### Overview

**Figure 1.**
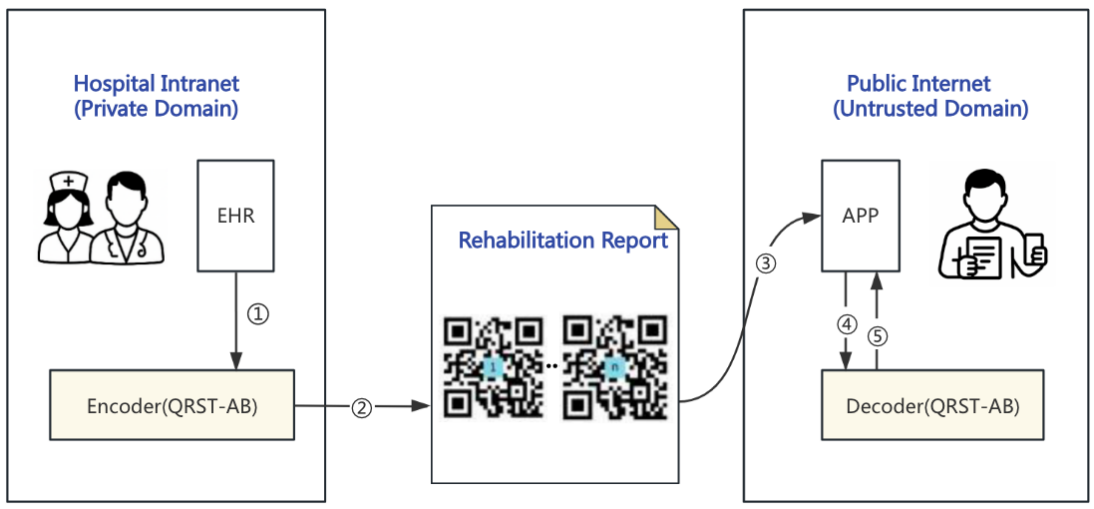
Application scenario of the proposed QRST-AB, illustrating the data flow of rehabilitation reports in cross-network environments. EHR: electronic health record; QRST-AB: QR code–based security transmission algorithm using Avro and byte pair encoding.

We propose a QR code–based secure transmission algorithm using Avro and byte pair encoding (BPE), which supports the secure creation and transfer of out-of-hospital health records for inpatients after discharge. The application scenario of the QR code–based secure transmission algorithm using Avro and byte pair encoding (QRST-AB), as illustrated in Figure 1, involves the following workflow: (1) health care providers access the patient’s EHR; (2) the encoder module applies the algorithm to generate one or more QR codes, which are embedded into a printed rehabilitation report and physically delivered to the patient; (3) the patient scans the QR code using a mobile app; (4) the app uploads the QR code content to a server-side module; and (5) the decoder performs verification and decryption, with results presented to the user. In this process, the encoder operates within the hospital intranet (trusted domain), whereas the decoder resides in the public internet (untrusted domain), enabling secure cross-network transmission.

#### Algorithm Framework

**Figure 2.**
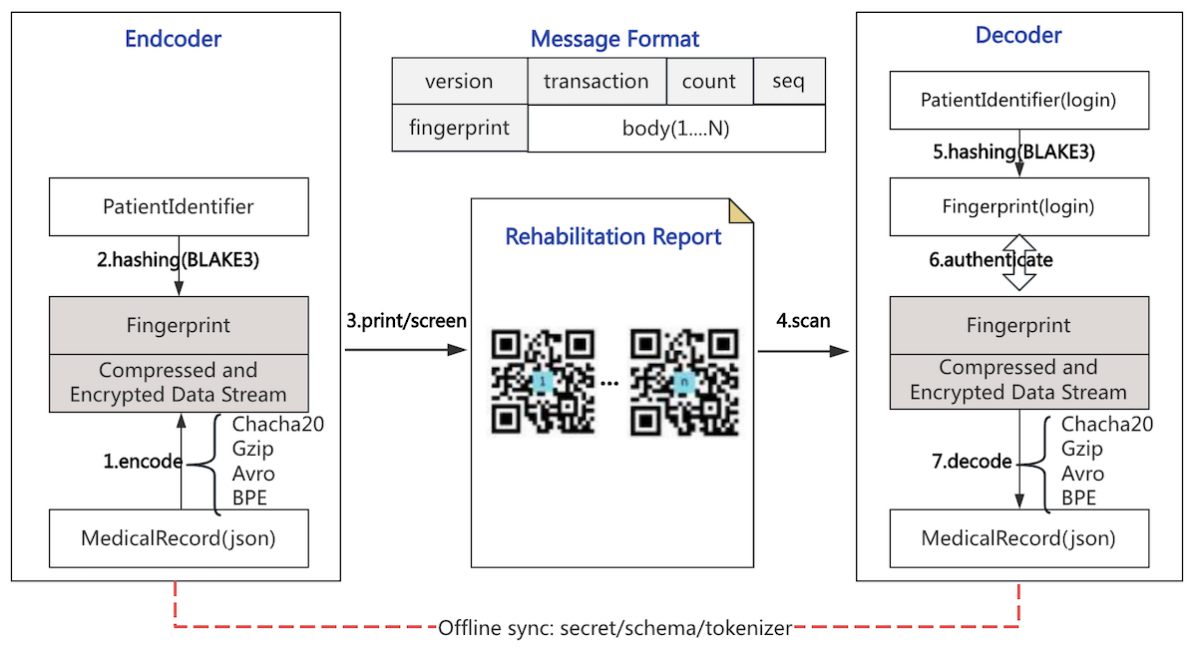
The framework and processing flow of the QRST-AB, illustrating how patient medical records are compressed, encrypted, split into QR codes, and securely transmitted and authenticated. BPE: byte pair encoding; JSON: JavaScript Object Notation; QRST-AB: QR code–based security transmission algorithm using Avro and byte pair encoding.

The QRST-AB algorithm efficiently compresses and encrypts patient medical records in JSON format, divides them into multiple QR codes for secure transmission, and authenticates users through encrypted digital fingerprints, ensuring privacy and data security. The algorithm consists of 2 components: an encoder and a decoder. The encoder and decoder periodically synchronize the secret, schema, and tokenizer through offline methods. The details of the framework and processing flow of the QRST-AB algorithm are shown in Figure 2 and Textbox 1.

**Textbox 1. The processing flow of the QR code–based secure transmission algorithm using Avro and byte pair encoding (BPE).**

1. The patient medical record is deidentified and separated into 2 parts: the patient identifier and the limited data set. The data is processed through a multistage encoding pipeline consisting of BPE, Avro, Gzip, and ChaCha20, to generate a compressed and encrypted data stream, which serves as the message body. The data stream is then divided into multiple subpackets (body1...N) based on the predefined maximum size of the QR code. BPE tokenizes and vectorizes the texts in the medical record; Apache Avro serializes the JSON (JavaScript Object Notation) objects into a binary format; Gzip compresses the binary data stream; Chacha20 encrypts the compressed data to ciphertext.
2. Every subpacket and patient identifier is hashed using the cryptographic function (BLAKE3) to generate a digital fingerprint and is encapsulated within the message header. This header is combined with body1..N to form multiple QR codes.
3. The QR codes are printed on A4 paper as part of the home rehabilitation report and handed to the patient, or displayed directly on a hospital computer screen for the patient to view.
4. The QR codes are scanned by a dedicated app on the smartphone, and the scanned content is uploaded to the server (decoder).
5. Upon receiving the QR code content, the decoder first generates a new digital fingerprint based on the logged-in user’s identifier.
6. Next, the calculated fingerprint is authenticated against the digital fingerprint in the QR codes, and only if the authentication passes, the decoding process is executed.
7. In the presence of multiple QR codes, a merging operation is conducted to form a complete information body, which is then converted back into a JSON object through the decoding process. The specific steps include the following: Chacha20 decrypts the ciphertext; Gzip decompresses the binary data; Avro deserializes the binary data back into a JSON object; and BPE restores the string representation of the JSON object from its numerical form.

#### Protocol Package Format

Within each QR code, the data content is divided into 2 parts: the message header and the message body. The header consists of 5 fields: version, fingerprint, transaction, count, and seq. The specific definitions of each field are shown in Table 2.

**Table 2.** Definition of the message header fields in each QR code of the QR code–based secure transmission algorithm using Avro and byte pair encoding.

Field	Definition	Value formula	Description	Len (bits)
Version	Version identifier, a predetermined fixed value	Constant	Used for compatibility with historical versions after updates.	8
Fingerprint	The message fingerprint	H(Message||IdentityID)|[1:n]	H() represents a cryptographic hash function. n represents the length of the digest in bytes, ranging from 1 to 64.	16
Transaction	Transaction ID for the current transmission	$T_{adm}+T_{tag}$	*T_adm_* represents the patient’s admission date, and *T_tag_* is a predefined date for a specific tag, such as ”2020-01-01”.	16
Count	Number of QR codes the record is split into	$\left[\frac{L_{body}}{L_{qr}-L_{header}}\right]$	*L_body_* is the total length of the compressed and encrypted body, *L_qr _*is the maximum length of a QR code, and *L_header_* is the fixed length of the message header	4
Seq	Sequence number of each QR code	$S_{prev}+1$	Starts from 0 and increments by 1.	4

#### Response Status Codes

When users receive information contained in the QR codes, the operation is simple—scanning each QR code sequentially using the dedicated app. The program provides friendly prompts based on the parsing results. The explanations for different response status codes are listed in Table 3.

**Table 3.** Response status codes and their explanations for users scanning QR codes with the dedicated app in the algorithm.

Result code	Explanation
finished	All QR codes have been successfully scanned, and decoding is complete.
waiting	Current QR code has been successfully scanned, please continue scanning others.
duplicated	Current QR code has been scanned repeatedly, please continue scanning others.
tag_error	Tag error, not a QR code format supported by the program.
auth_failed	Authentication failed, please verify authorization.
exception	Other exceptions, please contact technical support for assistance.

#### Algorithm Scalability

In this algorithm, each version can define the length of individual fields in the message header. Different versions correspond to different encoders, each with its own secret, schema, and tokenizer. On the decoder side, a multiversion manager enables support for various encoders, allowing compatibility with multiple data centers.

By leveraging the flexibility of the flexible structure of Avro Schema, it is easy to extend the system to support different JSON formats. In addition to the VTE electronic medical records discussed in this paper, the algorithm can be adapted to other disease types and even to nonmedical application scenarios, demonstrating strong scalability and versatility.

### Data Compression

#### JSON Serialization

JSON serialization refers to the process of converting data structures or objects into a string or binary form for the purpose of storage or transmission. The opposite process is deserialization, which restores the serialized data back to its original structure or object.

Within serialization methods, common types include schema-driven serialization and schema-less serialization. The former includes methods such as Abstract Syntax Notation One (ASN.1) using Packed Encoding Rules, Apache Avro, Microsoft Bond, Cap’n Proto, FlatBuffers, Protocol Buffers, and Apache Thrift. These methods rely on predefined schemas to ensure the structure and type of data, typically achieving higher space efficiency. Previous studies have shown that ASN.1 and Apache Avro perform particularly well in compression efficiency [15,16].

After comparative validation and in combination with the Gzip method, we found that Apache Avro outperforms ASN.1 in terms of compression efficiency, and Avro’s syntax definition is more compatible. Therefore, in our algorithm, we adopted Apache Avro as the serialization method.

#### Tokenization

Tokenization is a technique that converts text sequences into numerical sequences, serving as a foundational step in natural language processing tasks. It bridges the gap between raw text and language models. Existing tokenization methods, such as BPE, originate from the field of data compression. Some scholars believe that BPE is effective because it compresses text into fewer tokens, allowing the tokenizer to be trained more efficiently on specific datasets [17-19].

The prevalent tokenization techniques in the field of natural language processing include BPE, unigram, and WordPiece. These techniques are adopted by different large language models: BPE is used by the LLaMA series and GPT series, while unigram and WordPiece are used by bidirectional encoder representations from transformers and its variants. Given that our study focuses on Chinese medical texts, which lack explicit delimiters and cannot directly use WordPiece, we have selected BPE and unigram for comparing tokenization compression efficiency.

In our dataset, we compared the compression efficacy of 2 tokenization methods: BPE and the unigram. Experimental results indicated that the BPE method demonstrated superior compression performance within the algorithmic framework proposed in this study. Therefore, we ultimately selected the BPE tokenization strategy [20,21].

#### Data Compression Process

**Figure 3.**
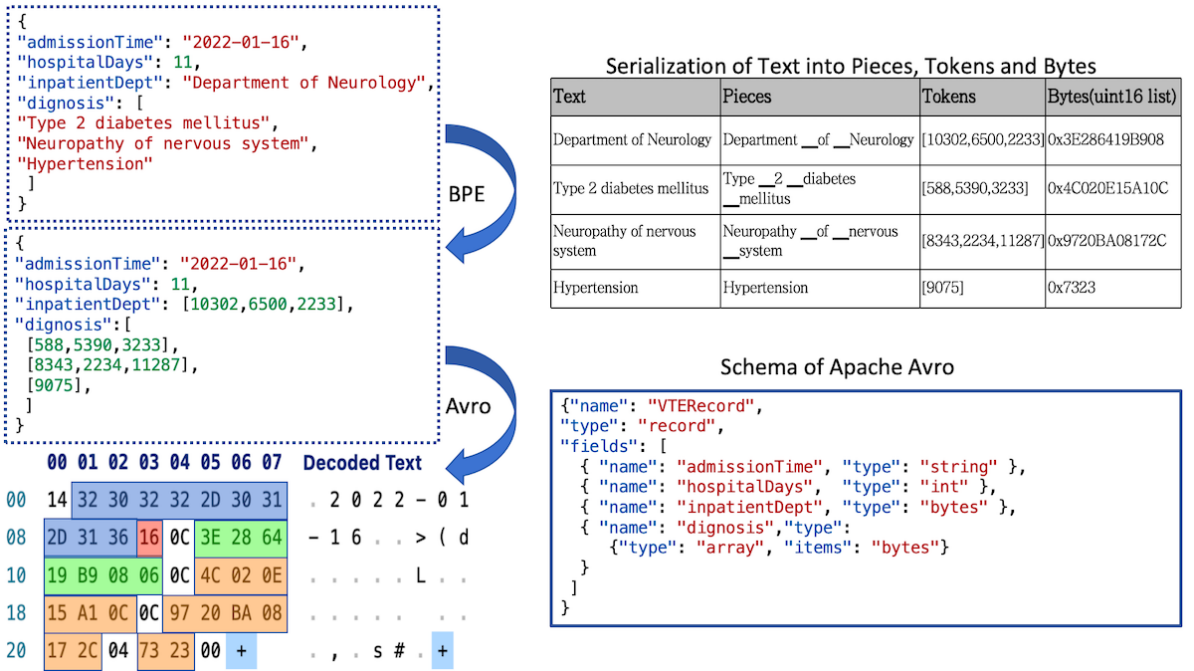
Explanation of the compression process using BPE and Avro, where BPE tokenizes using the tokenizer and Avro serializes JSON based on schema. BPE: byte pair encoding; JSON: JavaScript Object Notation.

To demonstrate the proposed data compression approach in practice, we present the following illustrative example using a simplified JSON record of patient information. The JSON file contains 4 fields: admissionTime, hospitalDays, inpatientDept, and diagnosis, as shown in Figure 3.

Tokenization is performed on 2 free-text fields: “inpatientDept” and “diagnosis”. For example, “Type 2 diabetes mellitus” is split by the tokenizer into “Type ▁2 ▁diabetes ▁mellitus” and further converted into a list of numerical values corresponding to tokens [10,302, 6500, and 2233]. It is noteworthy that due to differences in training corpora and hyper-parameter configurations, different tokenizers may generate varying segmentation results and tokens. This transformation not only contributes to compression but also provides a degree of information hiding, as the original text is not human-readable without the corresponding tokenizer.

Furthermore, Apache Avro is used to transform the JSON into a binary format to complete the serialization operation. As shown in the figure, the blue-marked bytes represent the value of the admission date “2022-01-16,” the red-marked bytes represent the length of hospital stay “11” (using variable-length zig-zag encoding, 11(integer)=22(zig-zag)=0×16(octet)), the green-marked bytes represent the hospital department, and the orange-marked bytes represent the 3 disease names of the diagnosis.

It is important to note that the Avro-encoded binary data does not contain type information or field names, which significantly reduces the size of serialized data. However, this also means that the receiving end must use the same schema as the sending end to correctly read the Avro data. In our algorithm, the Avro schema is not transmitted via the QR code, making it particularly difficult for third parties without the schema to read the Avro data.

In summary, the exclusive tokenizer and Avro schema definitions held by the server and client provide a robust defense for data security. In the example mentioned above, the original JSON data occupies 185 bytes, and after processing with the BPE tokenization and Avro serialization, the space is reduced to 38 bytes, with a compression ratio reaching 4.87, thereby demonstrating its excellent compression performance.

### Encryption and Authentication

Modern authenticated encryption adopts the authenticated encryption with associated data approach. National Institute of Standards and Technology recommends standard algorithms such as AES-GCM, AES-CBC+HMAC, and ChaCha20-Poly1305, which ensure both data confidentiality and message integrity [22]. However, these methods require additional overhead, including a 16-byte authentication tag and 8/12/24-byte Nonce or IV fields. AES-CBC further requires 16-byte block alignment, leading to significant message expansion that limits transmission efficiency in space-constrained QR codes.

This work adopts a symmetric authenticated encryption with associated data scheme, where both encoder (sender) and decoder (receiver) share a 256-bit secret key. ChaCha20 is used for encryption and BLAKE3 for authentication, resulting in only 2 bytes of overhead. ChaCha20 is an IETF-standardized stream cipher that uses a 256-bit key and 64-bit nonce, producing a keystream via 20 rounds of permutation. It does not require alignment, keeps the same ciphertext size, and works fast in software even without special hardware, making it suitable for mobile and low-latency scenarios. BLAKE3 is an advanced cryptographic hash function with higher performance than Secure Hash Algorithm 256 bit and Secure Hash Algorithm 512-bit. It supports variable output lengths, allowing flexible adaptation to different application requirements [23].

Compared with other transmission methods, QR codes printed on paper are harder to steal and are difficult to brute-force due to their low propagation efficiency. In our proposed algorithm, BLAKE3 is configured to produce a 2-byte hash, resulting in a brute-force probability of 1/(2^16), which is acceptable for practical authentication. ChaCha20 encryption, with a 256-bit key, offers a brute-force resistance of 1/(2^256), meeting the National Institute of Standards and Technology Level 3 security standard.

### Dataset

During the algorithm development phase, this study constructed a “Chinese Medical Text Dataset” for training a proprietary tokenizer. The dataset was sourced from the EHRs of approximately 80,000 historical patients from a hospital. The extracted fields included department names, surgical procedures, medication names, disease diagnoses, indicator names, and imaging examinations, among others, and were saved in text. The access control layer integrates time-based one-time password using a 6-digit random code, reducing the probability of unauthorized access to below $10^{-6}$.format.

In the algorithm validation phase, the study built a “Performance Benchmark Dataset” to verify the overall compression performance of the algorithm. This dataset was derived from the EHRs of 300 inpatients with VTE at the Sixth Medical Center of the Chinese PLA General Hospital between January and October 2024 and was saved in JSON format. These medical records comprehensively documented the patients’ basic information, surgical history, diseases, radiological examination results, laboratory test data, risk assessments, and medication upon discharge. Specifically, the basic information included the patient’s age, gender, height, weight, department, and length of hospital stay; surgical details recorded the name and timing of the surgery; disease classification encompassed the name and category of the diseases; radiological examination results detailed the date of the examination, the name of the procedure, the body part examined, and the conclusions; laboratory test data listed the name of the indicator, its value, unit, risk indication, and the normal range; medication upon discharge recorded the name of the medication, the daily dosage, and the frequency of administration; and the risk assessment section included the assessment scale, risk level, and risk factors.

### Performance Metrics and Validation Methods

The compression ratio in this study is defined by the formula: CompressionRate=OrigDataBytes/CompressedDataBytes.

In terms of algorithm design, we strive not only to optimize overall performance but also to ensure compatibility with various worst-case scenarios. Therefore, when analyzing the byte size of JSON files and the number of fields in the “Performance Benchmark Dataset,” we used statistical methods for calculating the mean, as well as the minimum and maximum extreme values.

We compared the efficiency of our compression algorithm with the traditional Gzip compression algorithm. In addition, we compared the efficiency of the 2 most effective JSON serialization technologies in real patient datasets, as well as the compression efficiency of 2 mainstream tokenization algorithms on specific datasets. All of the above comparisons used mean statistical methods and used *t* tests to calculate *P* values.

Furthermore, we used cumulative distribution function graphs to analyze the distribution of the number of QR codes scanned by patients and tested the time required for creating patient records via QR codes using various models of mobile phones.

### Ethical Considerations

This cohort study was approved by the Medical Ethics Committee of the Sixth Medical Center of Chinese People’s Liberation Army General Hospital (approval number: HZKY-PJ-2022-21). A waiver of informed consent was granted because this was a retrospective, data-only study, and all data were fully deidentified in accordance with institutional and national guidelines. Patients using the home rehabilitation service app provided informed consent at registration. QR code information constitutes a deidentified limited dataset with no personally identifiable information included; personally identifiable information is used only locally for authentication and is never transmitted. Encrypted QR codes are accessible only by authorized patients, ensuring data security and access control. All procedures complied with institutional policies, the Declaration of Helsinki, and relevant data protection regulations.

## Results

### Statistical Characteristics of the Performance Benchmark Dataset

We conducted a detailed statistical analysis of the fields and their storage sizes in the JSON files of the “Performance Benchmark Dataset,” which is summarized in Table 4. The average number of fields in the raw JSON files was 240.1, ranging from 89 to 623, with an average storage byte size of 7095, varying from 2748 to 17,425. In terms of the number of fields, laboratory test data constituted the highest proportion at 67.5%, followed by risk assessment data at 9.6%. Regarding storage byte size, laboratory test data also represented the largest proportion at 61.4%, with radiological examination result data following at 14.4%.

**Table 4.** Characteristics of the performance benchmark dataset in this study.

Patient (n=300)	Field (num), mean (min-max)	Field (%)	Size (bytes), mean (min-max)	Size (%)
Total	240.1 (89-623)	—^a^	7095 (2748-17,425)	—
Base	10 (10-10)	4.2	261 (242-270)	3.7
Operate	1.1 (0-32)	0.5	77 (23-1269)	1.1
Image	17.1 (4-56)	7.1	1022 (153-3944)	14.4
Lab	162 (46-496)	67.5	4354 (1269-13,365)	61.4
Ass	23 (10-35)	9.6	584 (212-1181)	8.2
Disease	12.2 (2-48)	5.1	419 (80-1537)	5.9
Drug	14.6 (0-85)	6.1	379 (28-2042)	5.3

a Not applicable.

### Overall Compression Efficiency Analysis

**Figure 4.**
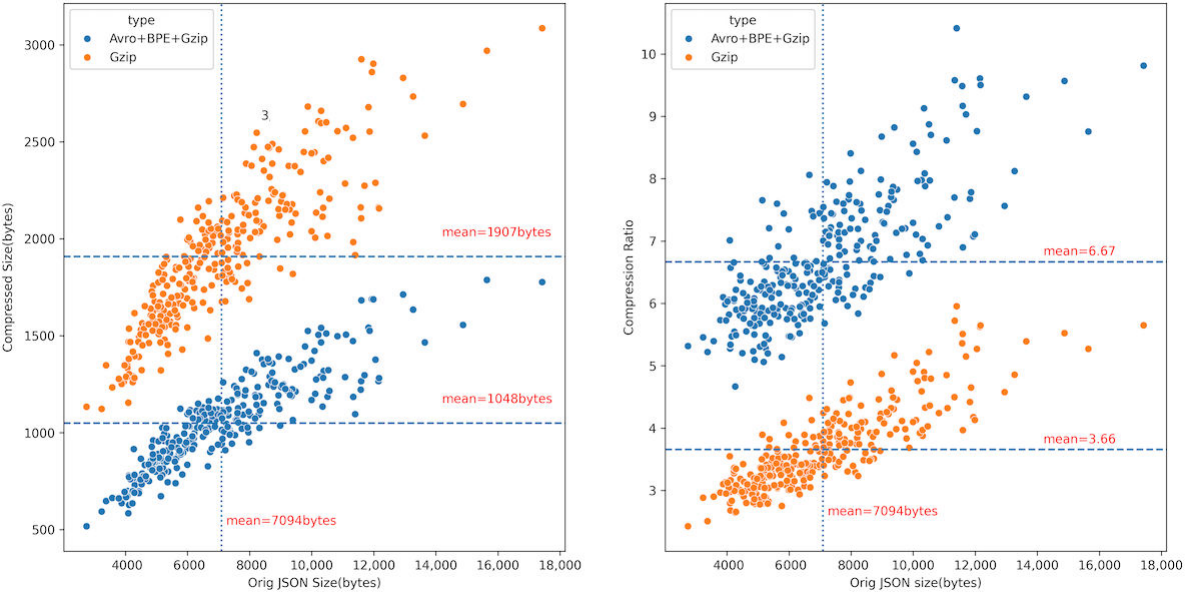
Comparison of the compression efficiency between our Avro + BPE + Gzip combined method and traditional Gzip in the performance benchmark dataset. BPE: byte pair encoding; JSON: JavaScript Object Notation.

The average size of the original JSON data was 7095 bytes. After compression using the traditional Gzip algorithm, the average size was reduced to 1907 bytes, with an average compression ratio of 3.66. By applying our developed BPE+Avro+Gzip combined compression algorithm, the average size was further reduced to 1048 bytes, improving the average compression ratio to 6.67. The compression efficiency of our algorithm was 1.82 times that of the traditional Gzip algorithm (*P*<.001). The scatter plot distribution in Figure 4 shows that the larger the original JSON file, the more significant the compression effect.

### Comparative Analysis of Avro and ASN.1

**Figure 5.**
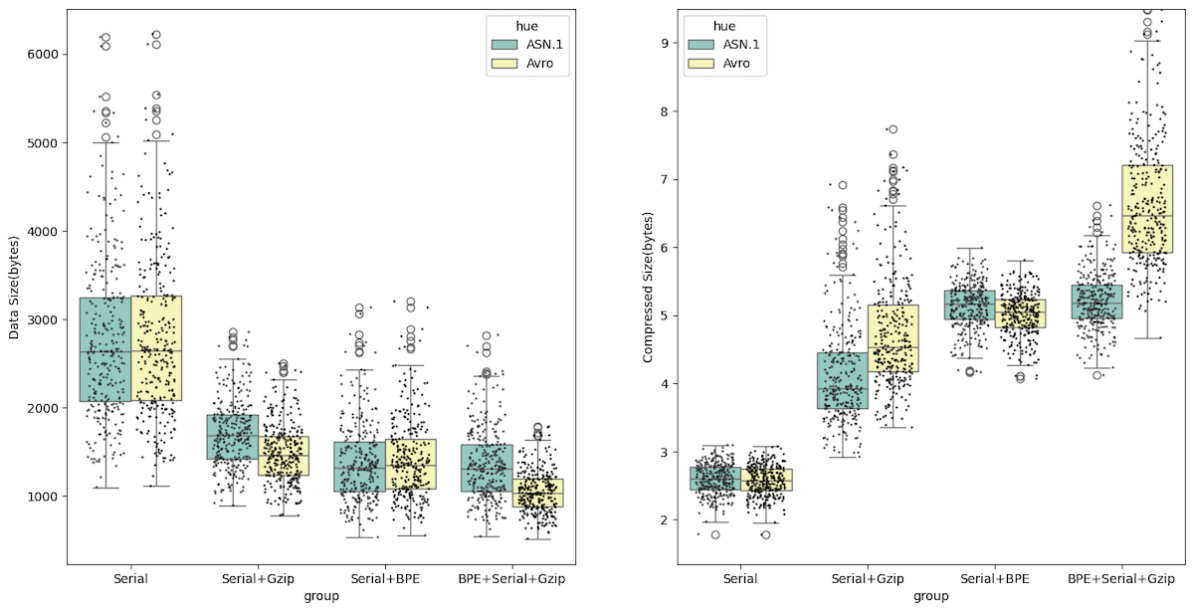
Analysis of compression performance for Avro and ASN.1 in different combinations with BPE and Gzip, where “Serial” indicates the use of Avro or ASN.1 for JSON serialization. ASN.1: Abstract Syntax Notation One; BPE: byte pair encoding; JSON: JavaScript Object Notation.

According to previous research, ASN.1 and Avro demonstrated the best compression performance among JSON serialization technologies. For our “Performance Benchmark Dataset,” Avro achieved a compression ratio of 2.59, while ASN.1 had a compression ratio of 2.57, with no significant difference (*P*=.30). However, Avro significantly outperformed ASN.1 when combined with BPE or Gzip methods (Table 5 and Figure 5). Specifically, the compression ratio for Avro+Gzip reached 4.72, compared with 4.12 for ASN.1+ Gzip (*P*<.001). Further, when the BPE method was introduced, the compression ratio for Avro+BPE+Gzip was 6.67, while for ASN.1+BPE+Gzip it was 5.21 (*P*<.001).

**Table 5.** Comparison of compression ratios between Avro and Abstract Syntax Notation One (ASN.1) in different combinations with byte pair encoding (BPE) and Gzip.

Name	Median (IQR)	Min	Max
ASN.1^a^	2.59 (2.45-2.77)	1.78	3.09
Avro	2.57 (2.43-2.75)	1.78	3.07
ASN.1+Gzip^b^	4.12 (3.63-4.46)	2.92	6.92
Avro+Gzip	4.72 (4.18-5.15)	3.35	7.73
ASN.1+BPE^c^	5.01 (4.83-5.23)	4.07	5.81
Avro+BPE	5.14 (4.94-5.37)	4.16	5.99
ASN.1+BPE+Gzip^d^	5.21 (4.95-5.45)	4.12	6.62
Avro+BPE+Gzip	6.67 (5.93-7.20)	4.67	10.41

a ASN.1 versus Avro; *P*=.30.

b ASN.1+Gzip versus Avro+Gzip; *P*<.001.

c ASN.1+BPE versus Avro+BPE; *P*<.001.

d ASN.1+BPE+Gzip versus Avro+BPE+Gzip; *P*<.001.

### Comparative Analysis of BPE and Unigram

Our constructed dataset consisted of a total of 1,146,532 records, with a total size of 122.5 MB and an average sentence length of 38.3 Chinese characters, corresponding to an average byte length of 106.8. We divided this dataset into a training set and a test set at a ratio of 9:1.

Using Google’s open-source project SentencePiece for tokenizer training, we trained and validated 2 tokenization methods: unigram and BPE171819, with a vocab_size of 65,535, allowing each token to be represented by a 2-byte unit. In terms of training duration, unigram took 65.91 seconds, while BPE took 360.2 seconds, with unigram being faster. In terms of compression ratio, on the training set, unigram and BPE achieved compression ratios of 4.7 and 4.83, respectively (*P*<.001); on the test set, they achieved 4.55 and 4.68, respectively (*P*<.001), showing that BPE is more efficient than unigram (Table 6).

**Table 6.** Comparison of compression ratios for byte pair encoding (BPE) and unigram with different serialization methods in the Chinese medical text dataset.

Dataset	Sentences (n=1,146,532)	Unigram	BPE	*P* value
Train	1,031,697	4.70	4.83	<.001
Test	114,835	4.55	4.68	<.001

### QR Code Distribution and Scanning Time

Based on previous research, when each module size is set to 4×4 pixels, 100% readability can be achieved on A4 white paper [24]. For the largest QR code size, the number of modules contained is 177×177 [25]. With a border width of 4 and an estimated resolution of 200 dots per inch for a standard printer, even the largest QR code would occupy a space of 3.7 inches (9.39 cm) in both length and width on A4 paper.

**Figure 6.**
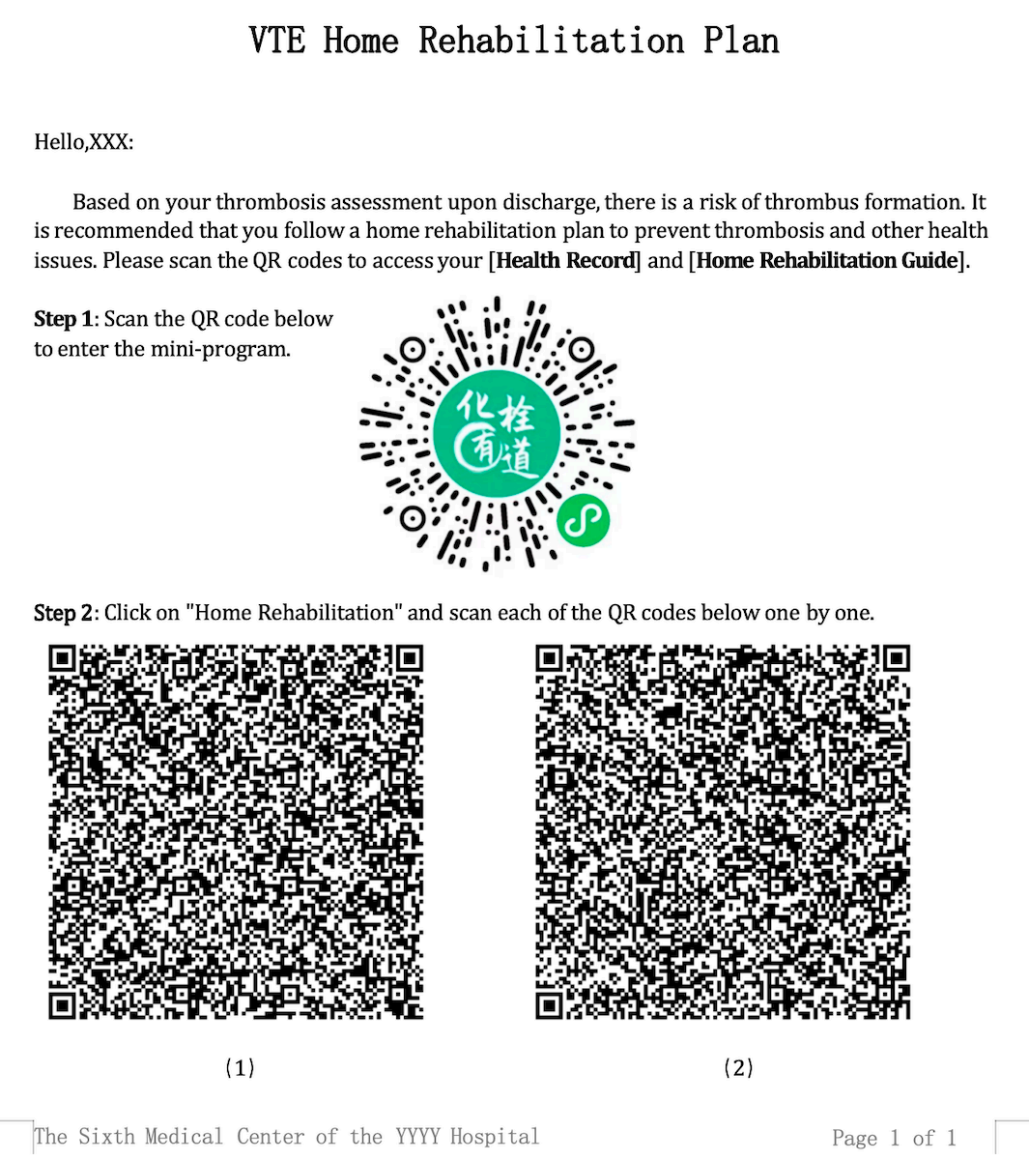
Example of a home rehabilitation report given to patients upon discharge for scanning QR codes. VTE: venous thromboembolism.

Considering the response speed of mobile phone scanning and the redundancy requirements of QR codes, we selected the version 25 QR code with 117×117 modules, which has a theoretical size of 2.5 inches (6.35 cm). Taking into account the differences in dots per inch, ink density, and default print margins across different hospital printers, we reserved a 20% redundancy, resulting in a designed QR code size of 7.62 cm × 7.62 cm (approximately 3×3 inches), with 2 QR codes per row. The specific layout is shown in Figure 6.

**Figure 7.**
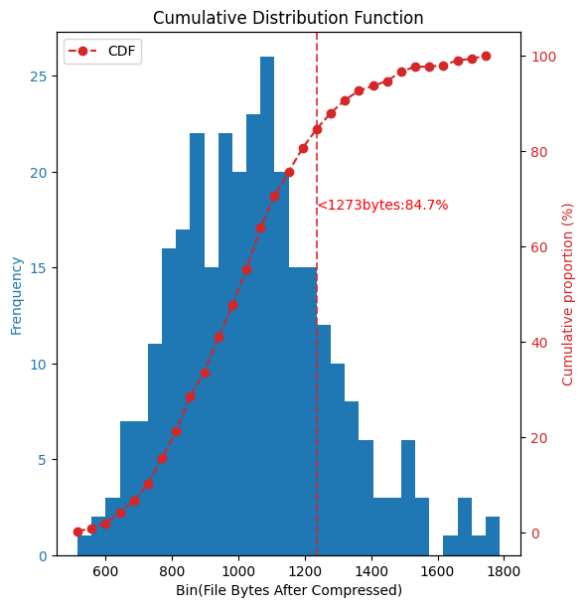
CDF analysis of file sizes for 300 patients after compression, showing that 84.7% of patients require scanning only 1 QR code with a file size less than 1273 bytes. CDF: cumulative distribution function.

We used version 25 QR codes with an error correction level set to L, each supporting a maximum binary capacity of 1273 bytes [25]. As shown in Figure 7, following a cumulative distribution function [26] analysis of the compressed file sizes for 300 patients, we found that 84.7% of the patients needed to scan only one QR code, while 5.3% of the patients required scanning 2 QR codes. Only an extremely small number of patients may need to scan more than 3 QR codes.

Based on the size of the compressed files, we selected 3 patients for a scanning test, corresponding to 1, 2, and 3 QR codes, respectively. The test timing started when the patient clicked the scan button and ended when the record creation was completed. Testing with different models of mobile phones showed that, on average, scanning 1 QR code took 3.1 seconds, scanning 2 QR codes took 7.30 seconds, and scanning 3 QR codes took 8.73 seconds. ANOVA was used to test the differences, with a *P* value <.001.

Based on the size of the compressed files, we selected 3 patients for a QR code scanning test, corresponding to 1, 2, and 3 QR codes, respectively. The timing started when the patient clicked the scan button and ended when the registration was completed. Different smartphone models were used for the tests, and the results showed that, on average, scanning one QR code took 3.1 seconds, scanning 2 QR codes took 7.30 seconds, and scanning 3 QR codes took 8.73 seconds (Table 7). ANOVA was used to test the differences, with a *P* value <.001.

**Table 7.** Duration (in seconds) for different types of patients to scan QR codes and complete record creation using various mobile phone models.

Phone model	Patient 1 (1 QR code)	Patient 2 (2 QR codes)	Patient 3 (3 QR codes)
iPhone 13 Pro Max	2.43	8.02	6.32
Xiaomi 13 ultra	2.18	5.95	8.79
iPhone 15 Pro	1.63	5.44	5.68
OPPO Find X5 Pro	1.94	6.50	7.70
OPPO K9s	6.03	8.83	10.54
Redmi K60	2.36	8.88	10.29
Xiaomi 10	2.98	6.28	9.37
Redmi Note 13 Pro	5.30	8.48	11.17
Mean	3.10	7.30	8.73

## Discussion

### Main Findings

We developed a QRST-AB, which assists patients in creating home rehabilitation records after discharge while ensuring the privacy, security, and efficiency of medical data transmission. The primary outcomes of our study are as follows. (1) We proposed a QR code authentication and encryption mechanism that leverages ChaCha20 encryption and the BLAKE3 hash function, reducing message inflation to as low as 2 bytes and enabling noninteractive cross-domain verification and data security in a zero-trust environment. (2) We developed an efficient combined data compression method, where the BPE+Avro+Gzip compression approach is 1.9 times more efficient than traditional Gzip compression, allowing a single patient’s medical record to be compressed into 1‐3 QR codes, thus improving the practicality and efficiency of QR code transmission in medical data scenarios. (3) The algorithm is provided in software development kit form, making it easy to deploy and use, which facilitates broader adoption.

### Comparison With Prior Work

In previous research, there have been instances of embedding medical data, as opposed to URL information, into QR codes for transmission. Specifically, Lin et al [8] encoded patients’ prescription information into QR codes, facilitating the use of these codes by patients at different pharmacies. The QR code encompassed information across 17 fields, including the patient’s name, identification, age, type of disease, name of medication, and date, among other critical data. Additionally, Nakayama from Japan [9] and Mathivanan from India [10,11] have successfully embedded electrocardiogram data into QR codes. Lauriot [12] adopted the approach of directly embedding the data results of imaging reports into a single QR code. Mao et al [13], on the other hand, used a different strategy; they segmented a file containing medical data and embedded the segmented data into consecutive QR codes, which were dynamically and continuously displayed in a streaming video. Users could capture this video stream with a smartphone and subsequently recover the relevant medical data from the QR codes.

However, in previous practices, despite the use of signal sampling and private encryption algorithms to enhance security when embedding electrocardiogram data into QR codes, medical texts are still embedded in plaintext, which may expose the data to interception by unauthorized third parties, leading to potential leakage of patient privacy information. Moreover, these cases generally lack user authentication mechanisms and exhibit low compression efficiency.

Prior to this work, several studies have proposed solutions for securing data transmission in untrusted environments. For example, [27] introduces authentication and encryption measures for military communication protocols to prevent man-in-the-middle and replay attacks. In [28], blockchain technology is used to establish a decentralized trust mechanism—for instance, similar to the framework described in [29], one can refer to its on-chain authentication and consensus processes to enhance data integrity and immutability when transmitting QR-embedded information. In the health care domain, [30] emphasizes the need to balance data security and privacy protection in telemedicine interoperability, which informs our design of QR code data encryption and client authentication. Moreover, [31] addresses deepfake disinformation strategies from a holistic cybersecurity perspective, offering guidance on ensuring the integrity and trustworthiness of data embedded in QR codes. Finally, [32] presents a hesitant fuzzy decision-making approach for usable-security assessment, which can be applied to optimize the usability of our underlying authentication workflow.

To address these security risks and efficiency issues, we have taken the following improvement measures. First, we have proposed a complete solution and an end-to-end communication mechanism, defined the protocol specifications, and added authentication information to the protocol header. We use cryptographic hash algorithms to verify the identity of the scanning user, preventing unauthorized users from scanning the QR code. Additionally, we use encrypted transmission, which remains secure even if a third party understands the principles of our algorithm, as they do not possess the ChaCha20 secret key, the Avro encoding schema, and the BPE tokenizer, and thus cannot decipher the contents of the QR code.

Second, we have proposed an efficient compression algorithm for patient medical record data, organically integrating JSON serialization technology, tokenization techniques based on large language models (including subword tokenization methods like BPE), and binary data compression technology. The combination of BPE+Avro+Gzip has achieved optimal compression performance. To date, there is no precedent for compressing electronic medical records using this combination. Although the transmission efficiency of QR codes is inherently limited and slow, our effective compression algorithm can significantly reduce the size of medical record data, thus greatly enhancing the practicality of QR codes in the transmission of medical information. Furthermore, our solution supports the transmission of multiple QR codes in a single transaction, thereby enabling the carriage of larger volumes of medical records and information.

Through our improvements and real-world data validation, the QRST-AB possesses the characteristics of encryption, authentication, efficiency, robustness, and scalability. This makes it well-suited for home rehabilitation registration scenarios and other situations requiring the transmission of patient privacy data.

### Limitations

There were some limitations to this study. First, due to the limited project timeline, validation was conducted using single-center data only. However, the algorithm was designed with flexibility for multicenter deployment and support for diverse disease types, which will be further validated in future studies. Second, when patient records are large and QR codes contain substantial data, poor network conditions may lead to delays in the decoder’s real-time response. Thirdly, the solution relies on manual scanning, and the patient’s digital health literacy may affect the user experience; however, most users are capable of performing scanning operations, which helps mitigate this issue.

### Future Work

We plan to conduct multicenter validation and extend support to other disease types, as well as optimize the algorithm to reduce response delays caused by large patient data volumes.

### Conclusions

The QRST-AB algorithm efficiently compresses and transmits data in an encrypted manner and authenticates the identity of the scanning users, ensuring the privacy and security of medical data. Delivered as a software development kit, the algorithm offers straightforward implementation and usability, supporting its broad adoption across various applications. All code is publicly available through the QRST-AB GitHub repository.

## Abbreviations

ASN.1: Abstract Syntax Notation One
BPE: byte pair encoding
EHR: electronic health record
JSON: JavaScript Object Notation
QRST-AB: QR code-based security transmission algorithm using Avro and byte pair encoding
VTE: venous thromboembolism

## References

[1] Heit JA (2015). Epidemiology of venous thromboembolism. Nat Rev Cardiol.

[2] Nicholson M, Chan N, Bhagirath V, Ginsberg J (2020). Prevention of venous thromboembolism in 2020 and beyond. J Clin Med.

[3] Mlačo A, Mlačo N, Bejtović D, Spužić M, Džubur A, Begić E (2020). Provoked venous thromboembolism during ten-year follow up at the Clinical Centre University of Sarajevo. Med Glas (Zenica).

[4] Hospital Pharmacy Professional Committee of the Chinese Pharmaceutical Association (2024). Chinese expert consensus on home management of oral anticoagulants. Natl Med J China.

[5] Wright A, McGlinchey EA, Poon EG, Jenter CA, Bates DW, Simon SR (2009). Ability to generate patient registries among practices with and without electronic health records. J Med Internet Res.

[6] Batra P, Phalnikar N, Kurmi D, Tembhurne J, Sahare P, Diwan T (2024). OCR-MRD: performance analysis of different optical character recognition engines for medical report digitization. Int J Inf Tecnol.

[7] Ujiie S, Yada S, Wakamiya S, Aramaki E (2020). Identification of adverse drug event-related Japanese articles: natural language processing analysis. JMIR Med Inform.

[8] Lin CH, Tsai FY, Tsai WL, Wen HW, Hu ML (2012). The feasibility of QR-code prescription in Taiwan. J Clin Pharm Ther.

[9] Nakayama M, Shimokawa H (2013). Evaluation of an electrocardiogram on QR code. Stud Health Technol Inform.

[10] Mathivanan P, Edward Jero S, Ramu P, Balaji Ganesh A (2018). QR code based patient data protection in ECG steganography. Australas Phys Eng Sci Med.

[11] Mathivanan P, Ganesh AB, Venkatesan R (2019). QR code–based ECG signal encryption/decryption algorithm. Cryptologia.

[12] Lauriot Dit Prevost A, Bentegeac R, Dequesnes A (2022). “Re-Materialized” medical data: paper-based transmission of structured medical data using QR-Code, for medical imaging reports. Stud Health Technol Inform.

[13] Mao H, Chi C, Yu J, Yang P, Qian C, Zhao D (2019). QRStream: a secure and convenient method for text healthcare data transferring. Annu Int Conf IEEE Eng Med Biol Soc.

[14] Bhardwaj C, Garg H An approach for enhancing data storage capacity in quick response code using zip compression technique.

[15] Viotti JC, Kinderkhedia M (2022). A survey of JSON-compatible binary serialization specifications. arXiv.

[16] Jackson S, Cummings N, Khan S (2024). Streaming technologies and serialization protocols: empirical performance analysis. arXiv.

[17] Delétang G, Ruoss A, Duquenne PA (2023). Language modeling is compression. arXiv.

[18] Gage P (1994). A new algorithm for data compression. C Users J.

[19] Schmidt CW, Reddy V, Zhang H (2024). Tokenization is more than compression. arXiv.

[20] Sennrich R, Haddow B, Birch A Neural machine translation of rare words with subword units.

[21] Kudo T Subword regularization: improving neural network translation models with multiple subword candidates.

[22] Nir Y, Langley A (2018). ChaCha20 and poly1305 for IETF protocols.

[23] Pandya M (2024). Performance evaluation of hashing algorithms on commodity hardware. arXiv.

[24] Tarjan L, Šenk I, Tegeltija S, Stankovski S, Ostojic G (2014). A readability analysis for QR code application in a traceability system. Comput Electron Agric.

[25] (2024). Information capacity and versions of the QR code. QRcode.com.

[26] (2024). Cumulative distribution function. Wikipedia.

[27] Kumar R, Khan RA (2024). Securing communication protocols in military computing. Netw Secur.

[28] Kumar R, Ahmad Khan R (2024). Securing military computing with the blockchain. Comput Fraud Secur.

[29] Sahu K, Kumar R (2024). A secure decentralised finance framework. Comput Fraud Secur.

[30] Sahu K, Kumar R (2025). Telemedicine: how to achieve interoperability without compromising data security. Br J Healthc Manag.

[31] Kumar R, Khan SA, Alharbe N, Khan RA (2024). Code of silence: cyber security strategies for combating deepfake disinformation. Comput Fraud Secur.

[32] Kumar R, Baz A, Alhakami H (2020). A hybrid model of hesitant fuzzy decision-making analysis for estimating usable-security of software. IEEE Access.

